# Demonstrating GWP*: a means of reporting warming-equivalent emissions that captures the contrasting impacts of short- and long-lived climate pollutants

**DOI:** 10.1088/1748-9326/ab6d7e

**Published:** 2020-04-02

**Authors:** John Lynch, Michelle Cain, Raymond Pierrehumbert, Myles Allen

**Affiliations:** 1 Department of Physics, University of Oxford, United Kingdom; 2 Environmental Change Institute, University of Oxford, United Kingdom; john.lynch@physics.ox.ac.uk

**Keywords:** climate change, carbon dioxide equivalent, carbon dioxide warming equivalent, global warming potential, GWP*, methane

## Abstract

The atmospheric lifetime and radiative impacts of different climate pollutants can both differ markedly, so metrics that equate emissions using a single scaling factor, such as the 100-year Global Warming Potential (GWP_100_), can be misleading. An alternative approach is to report emissions as ‘warming-equivalents’ that result in similar warming impacts without requiring a like-for-like weighting per emission. GWP*, an alternative application of GWPs where the CO_2_-equivalence of short-lived climate pollutant emissions is predominantly determined by changes in their emission rate, provides a straightforward means of generating warming-equivalent emissions. In this letter we illustrate the contrasting climate impacts resulting from emissions of methane, a short-lived greenhouse gas, and CO_2_, and compare GWP_100_ and GWP* CO_2_-equivalents for a number of simple emissions scenarios. We demonstrate that GWP* provides a useful indication of warming, while conventional application of GWP_100_ falls short in many scenarios and particularly when methane emissions are stable or declining, with important implications for how we consider ‘zero emission’ or ‘climate neutral’ targets for sectors emitting different compositions of gases. We then illustrate how GWP* can provide an improved means of assessing alternative mitigation strategies. GWP* allows warming-equivalent emissions to be calculated directly from CO_2_-equivalent emissions reported using GWP_100_, consistent with the Paris Rulebook agreed by the UNFCCC, on condition that short-lived and cumulative climate pollutants are aggregated separately, which is essential for transparency. It provides a direct link between emissions and anticipated warming impacts, supporting stocktakes of progress towards a long-term temperature goal and compatible with cumulative emissions budgets.

## 1. Introduction

A range of different climate pollutants contribute to anthropogenic climate change [1]. Emissions of different climate pollutants are often described using a common metric, generally scaled relative to carbon dioxide (CO_2_); hence non-CO_2_ emissions are typically communicated (and aggregated) as carbon dioxide equivalent (CO_2_-e) quantities.

The most widely used CO_2_ equivalence metric is the Global Warming Potential (GWP), defined as the integrated change in radiative forcing (the perturbation of the Earth’s atmospheric energy balance, which leads to warming) over a specified time-period following an emission pulse of a given climate pollutant, relative to the same quantity of CO_2_. Emissions of a given climate pollutant (*E*) can thus be converted to a CO_2_-e emission (${E}_{{{\rm{CO}}}_{{\rm{2}}} \mbox{-} e}$) quantity by multiplying by the appropriate GWP conversion factor, for the specified time-horizon (*H*):

1 \begin{eqnarray*}{E}_{{\mathrm{CO}}_{2} \mbox{-} {\rm{e}}}=E\times {\mathrm{GWP}}_{H}.\end{eqnarray*}

The 100-year variant of the Global Warming Potential (GWP_100_) has been formally adopted in international climate policy (currently as established in the Kyoto Protocol, and in the draft text of the Paris Agreement [2]) and standardised Life Cycle Assessment (LCA)/carbon-footprinting approaches [3]). Subsequently, GWP_100_ has become the *de facto* standard for expressing emissions in the scientific literature and general media, and has essentially become shorthand for the relative climate impacts of a given product or activity. Despite its ubiquity, the relationship between aggregate CO_2_-e emissions calculated using GWP_100_ and global warming itself is ambiguous.

This has been a significant and long-standing criticism of the GWP (e.g. [4–7]). Fundamentally, many of the shortcomings of the GWP as a universal climate metric arise because it cannot sufficiently differentiate the contrasting impacts of long- and short-lived climate pollutants (SLCPs), and it is this element that we focus on here.

A large fraction of anthropogenic CO_2_ emissions will persist in the atmosphere for millennia without active, large-scale efforts to remove them [8, 9]. Consequently, continued emissions add cumulatively to the atmospheric stock, and so within this millennial time period temperatures will increase indefinitely for as long as emissions are maintained, then remain approximately fixed at this level for centuries once emissions cease [10]. For greenhouse gases with relatively short atmospheric lifespans (or, more broadly, SLCPs) such as methane (CH_4_), however, natural atmospheric removals limit indefinite increases in their atmospheric concentrations for stable emission rates, as an equilibrium can be established where emissions and removals are approximately balanced. GWP_100_, or indeed any pulse-based metric treating long- and short-lived climate pollutants in the same way, cannot capture these contrasting dynamics.

Following these behaviours, sustained emissions of an SLCP therefore result in a similar impact to a one-off release of a fixed amount of CO_2_: both lead to a relatively stable long-term increase in radiative forcing. Thus an alternative means of equivalence can be derived, relating a change in the rate of emissions of SLCPs to a fixed quantity of CO_2_ ([7, 11–13], and see [14] for a recent elaboration on this equivalence concept). Allen *et al* [15] demonstrated that this can be achieved using GWP conversion factors (and thus based on the same basic atmospheric properties incorporated in the GWP), where a change in the emissions rate of an SLCP (Δ*E*_SLCP_) is equivalent to a one-off release or sequestration of ΔE_SLCP_ × GWP_*H*_ × *H* tonnes of CO_2_. This alternative application of GWPs is termed GWP*, and as the means of defining equivalence is better associated with temperature change contribution, can be considered a ‘CO_2_ warming equivalent (CO_2_-w.e.)’, in contrast to a per-emission CO_2_-e [16]. (For the rest of this letter we will use CO_2_-w.e. to report equivalents derived using GWP*, CO_2_-e for equivalents from conventional application of GWP_100_, and ‘CO_2_-equivalents’ as a generic term to describe either means of deriving equivalents). GWP* was further refined in Allen *et al* [17], showing that specifying a time-period (Δ*t*) of 20 years over which to assess the change in SLCP emission rates (Δ*E*_SLCP_), and scaling the CO_2_-w.e. per year of this period (i.e. /Δ*t*) provides a good fit for modelled warming, and a means of annualising the reported emissions.

Recent work by Cain *et al* [16] further improved the accuracy of GWP*. Sustained SLCP emissions result in stable forcing. Eventually, if maintained indefinitely, this results in no additional warming, but since most SLCP emission sources originated within the past century, there is a slow adjustment even to perfectly constant emissions due to the delayed response to past forcing increases [18]. For CO_2_, this slower temperature adjustment is approximately balanced by medium-term carbon cycle dynamics, particularly ocean CO_2_ absorption [19]. GWP* was consequently redefined to include a smaller component that also treats SLCPs as a ‘stock’ pollutant (similarly to conventional GWP_100_ usage) to account for this delayed response to past increases in SLCP emissions:

2 \begin{eqnarray*}\begin{array}{c}\begin{array}{c}\begin{array}{r}\begin{array}{c}{{E}_{{\rm{C}}{\rm{O}}}}_{2}-{\rm{w}}.{\rm{e}}.{}_{({\rm{S}}{\rm{L}}{\rm{C}}{\rm{P}})}=\left(r\times \displaystyle \frac{{\rm{\Delta }}{E}_{{\rm{S}}{\rm{L}}{\rm{C}}{\rm{P}}}}{{\rm{\Delta }}t}\times H\right.\\ \,\,+\,\left.s\times {E}_{{\rm{S}}{\rm{L}}{\rm{C}}{\rm{P}}}\right)\times {{\rm{G}}{\rm{W}}{\rm{P}}}_{H},\end{array}\end{array}\end{array}\end{array}\end{eqnarray*}

where *r* represents the weighting given to the impacts of changing the rate of SLCP emissions, and *s* the weighting given to the impacts of the current emissions rate.

These two weighting factors are scenario dependent (as they will vary based on the historical legacy of emissions, and hence how much of the slow warming component is already experienced), but using the GWP_100_ and a combination of *r* and *s* of 0.75 and 0.25 respectively was found to give a good approximation of the historical and projected warming impacts of methane over a range of emission trajectories [16], and we use these parameter values in this letter.

Using the values suggested above, under all scenarios except near-constant emissions the equation is dominated by (0.75 × 100 = 75) the rate-based component, with much less weight (0.25) assigned to the stock component. Both components are then multiplied by the GWP_100_ to provide a CO_2_ warming-equivalent quantity of emissions. Alternatively, if CO_2_-equivalent emissions are pre-computed using GWP_100_, they can be combined using the part of equation (2) in parentheses: hence GWP* is compatible with emissions reporting under the Paris Rulebook agreed at COP24, provided cumulative and SLCPs are reported and aggregated separately in emissions reporting and nationally determined contributions. The rule-book does not explicitly state that gases must be reported individually, although it is near-universal practice in reporting of emissions inventories to specify gases separately in terms of CO_2_-e. Separate reporting and aggregation of cumulative and short-lived pollutants in all communications between parties and the UNFCCC would substantially enhance the transparency of the UNFCCC process and ensure climatically important information is not lost.

With these suggested parameters, the GWP* equation can also be simplified further to:

3 \begin{eqnarray*}\begin{array}{c}\begin{array}{c}\begin{array}{ccl}{E}_{{{\rm{C}}{\rm{O}}}_{2}}-{\rm{w}}.{\rm{e}}.{}_{({\rm{S}}{\rm{L}}{\rm{C}}{\rm{P}})} &amp; = &amp; (4\times {E}_{\left.{\rm{S}}{\rm{L}}{\rm{C}}{\rm{P}}(t\right)}-3.75\\ &amp; &amp; \times \,{E}_{\left.{\rm{S}}{\rm{L}}{\rm{C}}{\rm{P}}(t-20\right)})\times {{\rm{G}}{\rm{W}}{\rm{P}}}_{100},\end{array}\end{array}\end{array}\end{eqnarray*}

where *E*_SLCP(*t*)_ is the current SLCP emission rate, and *E*_SLCP(*t* − 20)_ the rate of SLCP emissions 20 years ago, highlighting that GWP* requires only two values, which are already calculated and reported within the UNFCCC. This version of the equation can be thought of as representing that any ‘new’ methane emissions have a very strong climate impact, 4 times greater than reported by GWP_100_, but after 20 years much of the warming caused is automatically reversed.

Longer-lived climate pollutants (those with a lifespan longer than *H*, i.e. 100 years), will also display cumulative behaviours over this timeframe, and so their GWP* CO_2_-w.e. follows conventional use of GWP as in (1). Note, however, that this is in the context of formulating near-to-medium term climate policy, and is not meant to suggest that, for example, nitrous oxide (N_2_O) is directly equivalent to CO_2_ either; the impact of our emissions on the carbon cycle means that CO_2_ is unique, and the gas is uniquely long-lived [1, 7]. Whether a given climate pollutant is defined as short- or long-lived depends on the timescale being considered, and GWP* could potentially be applied using longer GWP time-horizons than 100 years to also treat longer-lived gases than methane as ‘short-lived’ (i.e. their impacts depend on the ongoing emissions rate, rather than cumulative total emissions, as for CO_2_). Further elaboration comparing different timeframes and climate pollutants is shown for related approaches, the combined global warming potential (CGWP) and combined global temperature-change potential (CGTP) in [14]. For this letter we focus exlusively on methane, as the most important SLCP, which must be treated as a non-cumulative pollutant to anticipate the impacts of ambitious emission reduction scenarios even over the next few decades.

Although relatively simple, this new means of deriving CO_2_-equivalence is a significant reframing of how we report and conceptualise emissions of SLCPs. It is worth illustrating and expanding upon the principles described above, and the nature of GWP*. In this study we demonstrate a number of methane and CO_2_ emissions scenarios in a simple climate model to display their contrasting dynamics. We show how these emissions scenarios translate to equivalents derived using either GWP_100_ or GWP* noting some of the shortcomings of GWP_100_, and how these are overcome by GWP*. We then highlight special cases and suggested applications that may provide extra insight or be of particular policy relevance.

## 2. Methods

We used the FaIR (Finite-Amplitude Impulse Response) v1.3 climate-carbon-cycle model to generate changes in atmospheric concentration, radiative forcing and temperature for a number of methane and/or CO_2_ emissions scenarios. FaIR captures the relevant dynamics of different climate pollutants, and has been shown to provide good agreement with other, more computationally intensive, climate modelling approaches [20]. We constrained methane to an average lifespan of 12 years to reflect contemporary conditions, and did not include variations in solar and volcanic forcing to focus on the impacts of our emissions scenarios alone. Radiative forcings are modelled following [21]. Our scenarios are based on perturbations from the standard RCP4.5 pathway from the year 2000. Default RCP4.5 emissions are generated from [20] to produce RCP4.5 concentrations [22] in FaIR. RCP4.5 was used to provide a ‘middle-of-the-road’ scenario, and with relatively stable methane emissions over time (discussed further below). The emissions specified for each scenario were added to these RCP4.5 defaults, and the difference between concentrations, forcings and warming from the modified scenarios and default RCP4.5 show individual contributions of the specified pathways alone.

Individual emission scenarios are described alongside their results below, but were broadly variations on a baseline scenario introducing and then sustaining a methane emission of 4 Mt per year. A default annual emission of 4 Mt methane, close to the average UK methane emissions between 1990 and 2016 [23], was selected to represent a significant, policy-relevant methane emission that would not be so large as to greatly perturb the RCP4.5 background conditions. For reference, this is just over 1% of the 353 Mt total anthropogenic methane emissions estimated for 2012 [24].

‘Equivalent’ CO_2_ emissions scenarios were derived for GWP_100_ and GWP* using (1) and (2) respectively, both with a GWP_100_ conversion factor for methane of 32 from [21]. A fixed GWP value was used, as though in practice this should be updated as greenhouse gas concentrations and hence the unit forcing per emission change in the future, most current climate policy is also based on fixed, present-date GWP values (see also below and supplementary material section 1, available online at stacks.iop.org/ERL/15/044023/mmedia for further discussion on this topic).

## 3. Results and discussion

### 3.1. Atmospheric behaviours of methane and CO_2_

**Figure 1.**
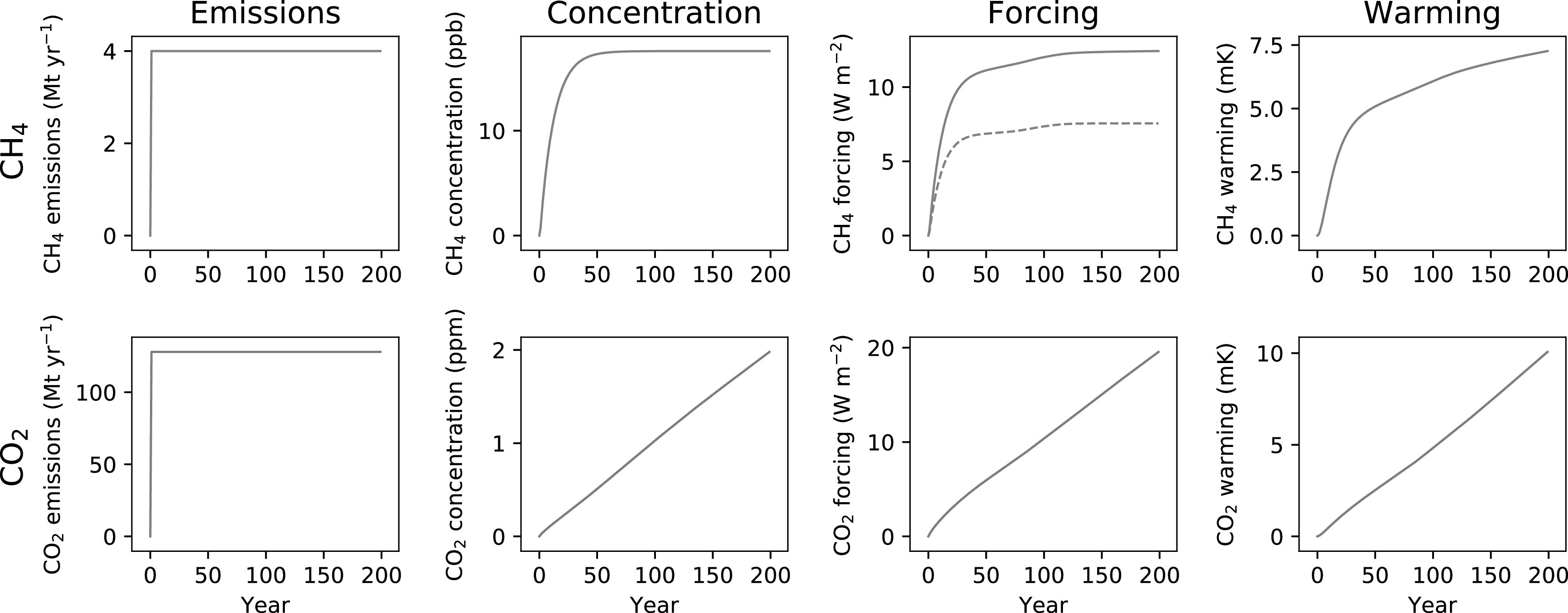
A step change to sustained emissions of CH_4_ (top row, orange) and CO_2_ (bottom row, purple), and resulting impacts on atmospheric concentration, radiative forcing and temperature. For CH_4_ forcing, the dotted line shows forcing from methane alone, but the total forcing impact (solid line), is greater than this as a result of the ozone and stratospheric water vapour produced as methane breaks down (total forcing is approximately 1.65 times that of methane alone [1]). Temperature change is modelled from total forcing.

To illustrate the principles outlined in the introduction, we start with a simple scenario where an emission is introduced in year 0 and then sustained at the same rate for 200 years (figure 1). For methane, concentrations increase rapidly but then plateau after a few decades as natural atmospheric removals balance ongoing emissions. Radiative forcing similarly increases rapidly due to the high radiative efficiency of CH_4_ (i.e. there is a large change in forcing per change in CH_4_ concentration) but then largely stabilises—although there is some increase in forcing between approximately years 50 and 150 as over this period the background methane concentration in RCP4.5 is expected to decline as anthropogenic emission rates are reduced, resulting in a greater radiative efficiency of any remaining methane emissions (i.e. including those modelled here).

This stabilising behaviour is then also reflected in the warming that results from the sustained CH_4_ emission: the bulk of the temperature change occurs rapidly, and is significant, but the rate of temperature increase declines after these initial decades. There is, however, still some long-term delayed adjustment to this initial increase. In the very long term (several centuries, but subject to the large uncertainties in the slow temperature equilibration of the Earth) the warming from any biogenic methane emission would also be expected to completely stabilise and generate no additional temperature increases, becoming indistinguishable from long-standing natural methane emission rates to which the climate system has fully adjusted [16], but to anticipate near- to medium-term climate impacts this extended temperature change remains important [18].

For CO_2_, modelling an equivalent (using GWP_100_) emissions scenario, we see that sustained emissions do not result in stabilising concentrations. Instead, concentrations, forcing and subsequently warming continue to increase for as long as the emissions are sustained, with an approximately linear relationship between cumulative CO_2_ emissions and resultant warming (as will be expanded upon later).

This simple illustration of the impacts of sustained emissions already highlights how different short- and long-lived GHGs are, and hence why metrics that attempt to treat them in the same way will fall short. These differences are even more pronounced if we consider the impacts of decreasing emission rates.

**Figure 2.**
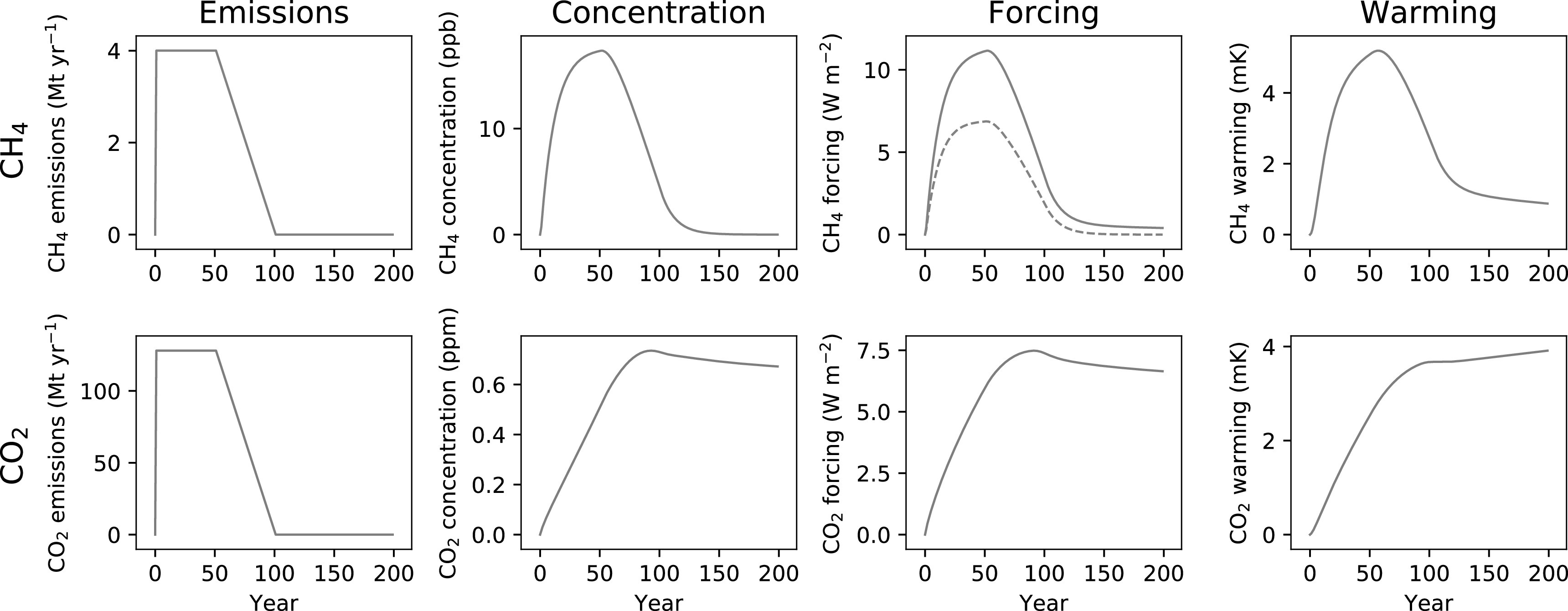
Impacts on atmospheric concentration, radiative forcing and temperature for a step change to sustained emissions for 50 years, followed by a decline to 0 emissions over the following 50 years for CH_4_ (top row, orange) and CO_2_ (bottom row, purple).

In figure 2 we demonstrate a scenario which begins in the same way as the previous example, with a step change to sustained emissions, but after 50 years these decline linearly to reach 0 over the following 50 years. For methane, as the emissions rate declines, so too do atmospheric concentrations: from the equilibrium described earlier, we start to reduce emissions while removals continue, and so atmospheric concentrations fall when they are not continuously ‘topped up’ by maintained emissions. These dynamics are then reflected in the change in forcing and ultimately warming, with declining emissions able to reverse a significant proportion of recent warming over relatively short timescales.

For CO_2_, reducing the emission rate merely acts to slow the rate of continued increase in concentration until emissions reach nearly 0, at which point atmospheric concentrations slowly decline as a result of short- and medium-term carbon sinks. It would take millennia for current CO_2_ concentrations to return to pre-industrial levels without active intervention [9, 7]. The changes in CO_2_ concentration are reflected in the change in forcing over time. Nonlinearities in forcing and warming approximately balance each other out such that the increased temperatures from CO_2_ emissions are relatively fixed at this amount once emissions cease [19], maintaining the near-linearity between cumulative CO_2_ emissions and warming noted earlier even without ongoing emissions.

A ‘zero emissions’ target will therefore have distinct impacts depending on whether the climate pollutant is long- or short-lived, and does not imply an equivalent mitigation effort or climate response.

The differing warming legacies once emissions of either gas are removed, as shown in figure 2, also applies to one-off emission pulses. Despite pulse-comparisons being commonplace in metric design (including the GWP) and individual emission offsets, trying to describe the impacts of a pulsed emission of CO_2_ with a scaled individual pulsed emission of methane, and vice versa, will inherently fail to provide similar warming pathways, and so transactions based on such exchanges will not result in climatically equivalent responses.

These two scenarios also highlight a broad principle: CO_2_-induced warming depends on cumulative emissions to date, but for SLCPs such as methane, their warming contribution is predominantly a function of recent emission rates [11, 25]. GWP* can capture this key difference.

### 3.2. CO_2_ equivalence of methane emissions

**Figure 3.**
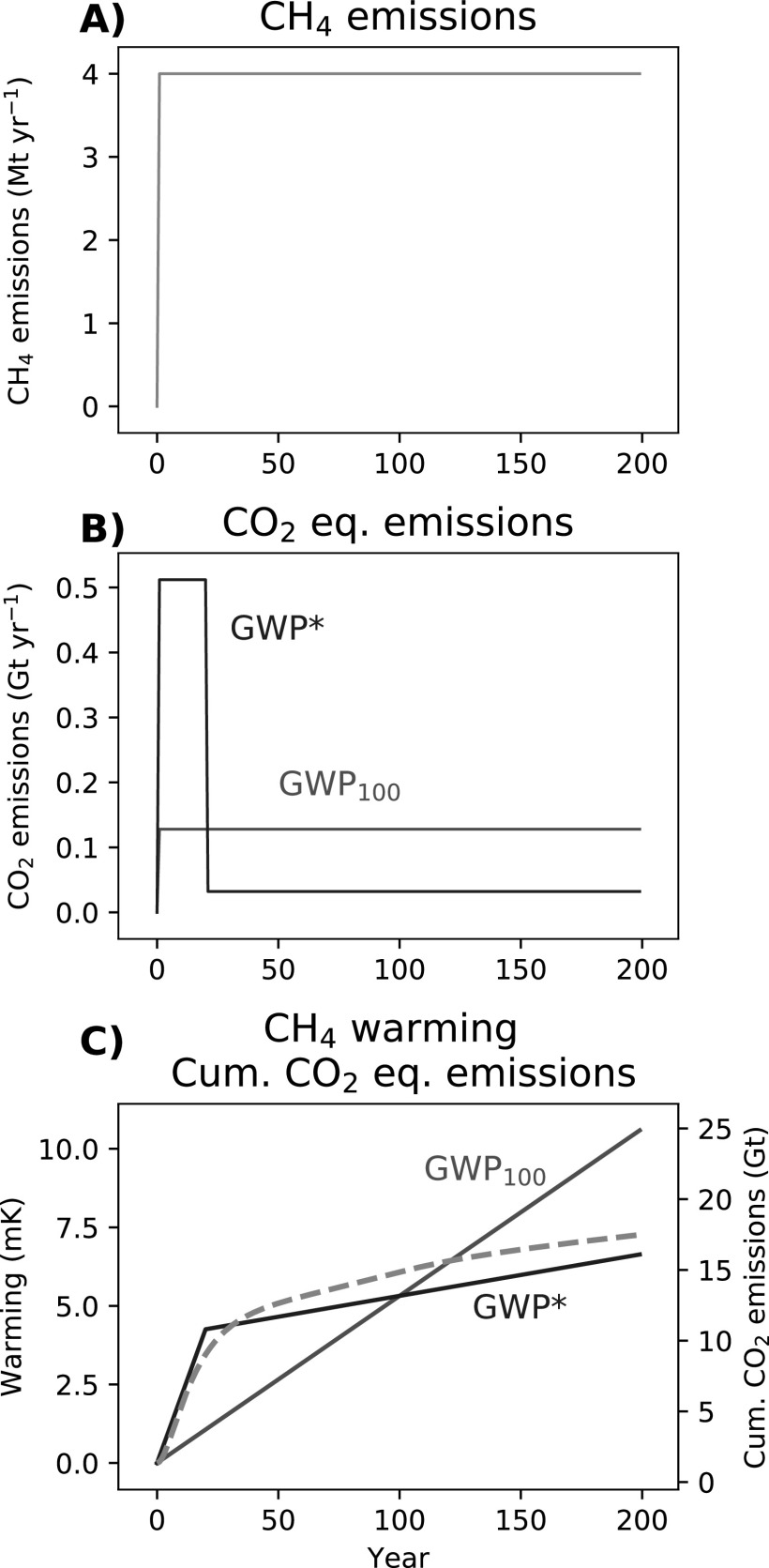
A demonstration of (A) a step-change to sustained CH_4_ emissions and (B) corresponding annual CO_2_-equivalent emissions using GWP_100_ or GWP* (red and blue lines, respectively), followed by (C) the warming resulting from those CH_4_ emissions (dashed orange line) overlaid with cumulative GWP_100_ and GWP* CO_2_-equivalent emissions (solid red and blue lines, respectively).

We now show how the corresponding GWP_100_ and GWP* CO_2_-equivalents compare for these two emissions scenarios. Starting with a step-change to sustained methane emissions (figure 3), using conventional application of GWP_100_ reports these methane emissions as equivalent to a constant rate of CO_2_-e emissions over time (this is also the same CO_2_ emissions scenario as shown in figure 1). Using GWP* over the first 20 years the methane emissions are represented by very high CO_2_-w.e. emissions, capturing the strong weighting given to the initial change in rate [from 0 emissions], but after this $\tfrac{{\rm{\Delta }}{E}_{{\rm{SLCP}}}}{{\rm{\Delta }}t}=0$, and so only the much lower CO_2_-w.e. emissions from the ‘stock’ behaviour of longer-term sustained methane emissions remain.

The main difference between GWP* and static metrics such as GWP_100_ is thus: with static metrics, individual emissions are directly equated by a single value that can only represent one particular impact at or over a stated time, and which cannot fully capture the temporal differences between the impacts of different gases; but for GWP*, ‘equivalent’ CO_2_ can vary in order to decribe dynamic responses over any time-frame of interest. As this problem occurs for any static concept of equivalence, it cannot be overcome by using alternative metrics or alternative time-horizons, as illustrated in section 2 of the supplementary material, revealing similar limitations for the 20 year Global Warming Potential (GWP_20_) and the 100-year Global Temperature change Potential (GTP_100_)

As described above, there is an approximately linear relationship between cumulative CO_2_ emissions and temperature. If CO_2_-equivalents do indeed describe methane emissions in an equivalent manner to CO_2_, a cumulative CO_2_-equivalent should therefore correspond to the warming generated by the methane emissions they describe. We can illustrate this by plotting our cumulative CO_2_-equivalents alongside temperature, as in figure 3(c). While GWP* provides a reasonable fit for the methane warming profile (initially rapid temperature increases followed by a longer tail of more gradual warming GWP_100_ suggests) a fixed rate of temperature change from methane that does not capture the true dynamics.

Directly relating equivalent emissions to temperature responses was not an intended usage of GWP_100_, and so attempting to link cumulative GWP_100_ CO_2_-e to global warming is arguably an inappropriate application of the metric. However, the approach provides useful insight into how the impacts of short-and long-lived gases differ, why static metrics such as the GWP_100_ will always miss important dynamics, and reveals further applications that become possible when the equivalent emissions reflect the impacts of cumulative CO_2_ emissions.

A simple coefficient, the TCRE (Transient Climate Response to cumulative carbon Emissions), can link cumulative CO_2_ emissions and temperature as a result of their linear relationship [26, 27]. Not only can cumulative CO_2_-w.e. provide an indication of the shape of the warming profile, threfore, but also suggest a direct correspondence between the scale of emissions and warming. This is represented by the alignment of the two *y* axes in figure 3(c) (and every figure hereafter combining temperatures and cumulative emissions), which are scaled by a TCRE of 0.4 K per Tt CO_2_ (1.52 K per Tt C). This TCRE was obtained by identifying the warming response to cumulative CO_2_ emissions for RCP4.5 within the same FaIR set-up described above (see supplementary material). By expressing short-lived GHGs as a CO_2_-w.e. consistent with a ‘cumulative carbon’ framework, GWP* thus provides a means of directly linking emissions of different gases to their resulting temperature impact through the TCRE.

GWP*, as a simple metric reliant on assumptions of linearity inherent in the GWP, cannot capture the full complexities of the climate response that alternative approaches may be able to, such as CO_2_-forcing-equivalents [25], the CGTP and CGWP [14], or comprehensive modelling of the climate system. Consequently, the match between cumulative CO_2_-w.e. emissions (×TCRE) and warming is not exact (and in this case the underestimation of warming may reflect, among other dynamics, an increase in the radiative efficiency of methane over this period expected as wider global methane concentrations fall under RCP4.5 [28]), while a fixed GWP was used here. There will remain applications where more complex methods are preferred, but the ease of calculating GWP* will likely prove a significant advantage for many purposes.

Both GWP_100_ and GWP* CO_2_-equivalents and the methane temperature response approximately correspond at 100 years (previously noted in [29, 30] and [31], observing that the sustained Global Temperature change Potential (sGTP)—that is, the temperature change following sustained emissions over a given period—is close to the GWP). This means that, looking back over 100-years of an introduced then sustained emission, GWP_100_ could also provide an appropriate indication of the total warming over this period; but otherwise can provide only limited inference. This correspondence is only the case for newly-introduced emissions subsequently sustained at the same rate over this period, and only at approximately 100 years: GWP_100_ would understate the total warming impact of sustained emissions before this point, and increasingly overstate the impacts after it. Even within this 100-year period, given that most of the warming occurs in the first few decades of the sustained methane emission, GWP_100_ will still overstate the annual contribution to warming at any point after this initial period. For example, if we are in year 30 of this scenario, the bulk of the warming that will result from this sustained methane emission is already experienced (the scale and speed of this warming greatly undervalued by GWP_100_), so from this point onwards GWP_100_ would provide an exaggerated estimate of how much additional warming to expect, and hence provide little insight in anticipating future temperatures increases (or associated climate impacts).

**Figure 4.**
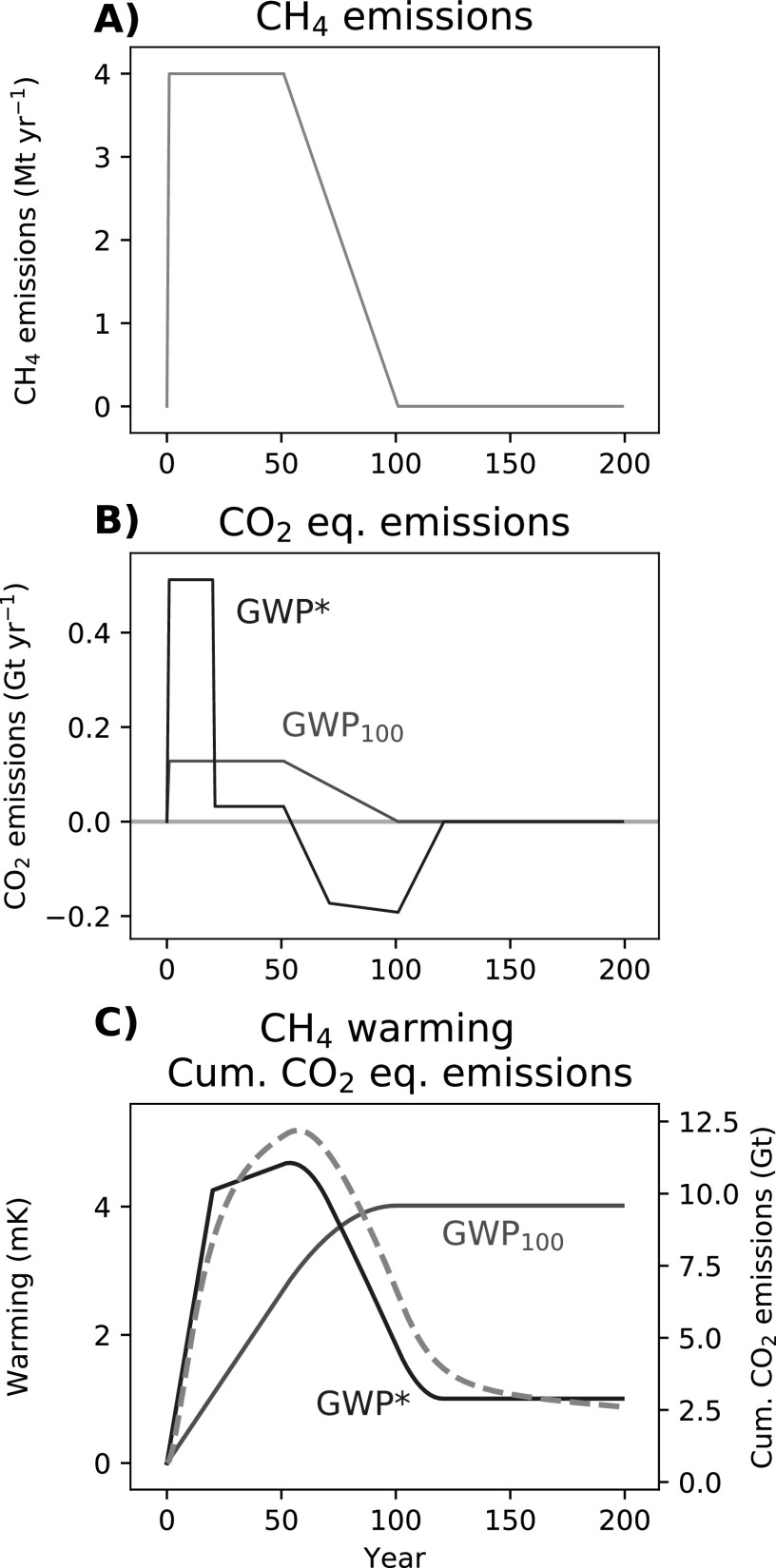
A demonstration of (A) a step-change to sustained CH_4_ emissions for 50 years followed by a decline to 0 emissions over the following 50 years, and (B) corresponding annual CO_2_-equivalent emissions using GWP_100_ or GWP* (red and blue lines, respectively), followed by (C) the warming resulting from those CH_4_ emissions (dashed orange line) overlaid with cumulative GWP_100_ and GWP* CO_2_-equivalent emissions (solid red and blue lines, respectively).

Once again, the scenario in which emissions start to decline further emphasises how differently the two gases behave (figure 4). As methane emissions decline, GWP_100_ CO_2_-e emissions also decline, but they stay positive (figure 4(b)), which, by comparison with the behaviour of CO_2_, can only indicate a slowing of the resultant warming until emissions reach 0 (as shown in figure 2), at which point the temperature would be expected to remain at this elevated level (figure 4(c)).

Using GWP*, the annual CO_2_-w.e. emissions profile is more complex (figure 4(b)), as the 20 year reference period tracks across various transitions in the methane emissions profile, but crucially, in the period of declining methane emissions CO_2_-w.e. emission are negative (i.e. representing a removal of CO_2_ from the atmosphere). Figure 4(c) shows how this is essential to capture the temperature impacts of this methane emissions pathway: the automatic reversibility of most methane warming once the emissions are reduced or removed can only be described by a negative CO_2_-equivalent, and so there is no real correspondence between the impact of this methane scenario and cumulative GWP_100_ CO_2_-e.

It must be emphasized that during the period of declining methane emissions, using GWP_100_ does not even indicate the correct direction of temperature change. The improvement achieved by using GWP* is therefore not simply a slight refinement to the shape of the warming profile or a better scaling of cumulative emissions to temperature under a given TCRE: it captures the fundamentally different behaviour of short- and long-lived climate pollutants, which is essential to provide even a broad indication of the impacts of ambitious mitigation strategies [32].

### 3.3. Key principles and special cases of GWP*

To further highlight some of the important elements described by GWP*, we now explore a number of shorter-term emission scenarios.

As noted above, GWP_100_ can variously over- or under-state the impact of methane emissions depending on the historic trajectory of these emissions, which may not be known. Here we show how application of GWP*, using the previous 20 years but no further, still gives a useful indication of anticipated temperature change. We also focus on a 50 year period to demonstrate that GWP* provides a much improved indication of short- and medium-term impacts, and is not relevant only for the longer (200 year) scenarios shown above.

Since most policy scenarios involve some prior methane emissions, we need to include these to show various dynamics that include decreases in emission rate, or incorporate behaviours that emerge in the longer term, and so all of the scenarios shown occur following 50 years of an introduced and then sustained methane emission, building on the examples above. We then change methane emissions to a new demonstrative trajectory, and after 25 years of this change, show the warming response and both CO_2_-equivalent emissions (hence these figures start from ‘year 75’). See supplementary material for further description of this approach, including an illustration in figure S4.

#### 3.3.1. Stable and declining methane emissions

**Figure 5.**
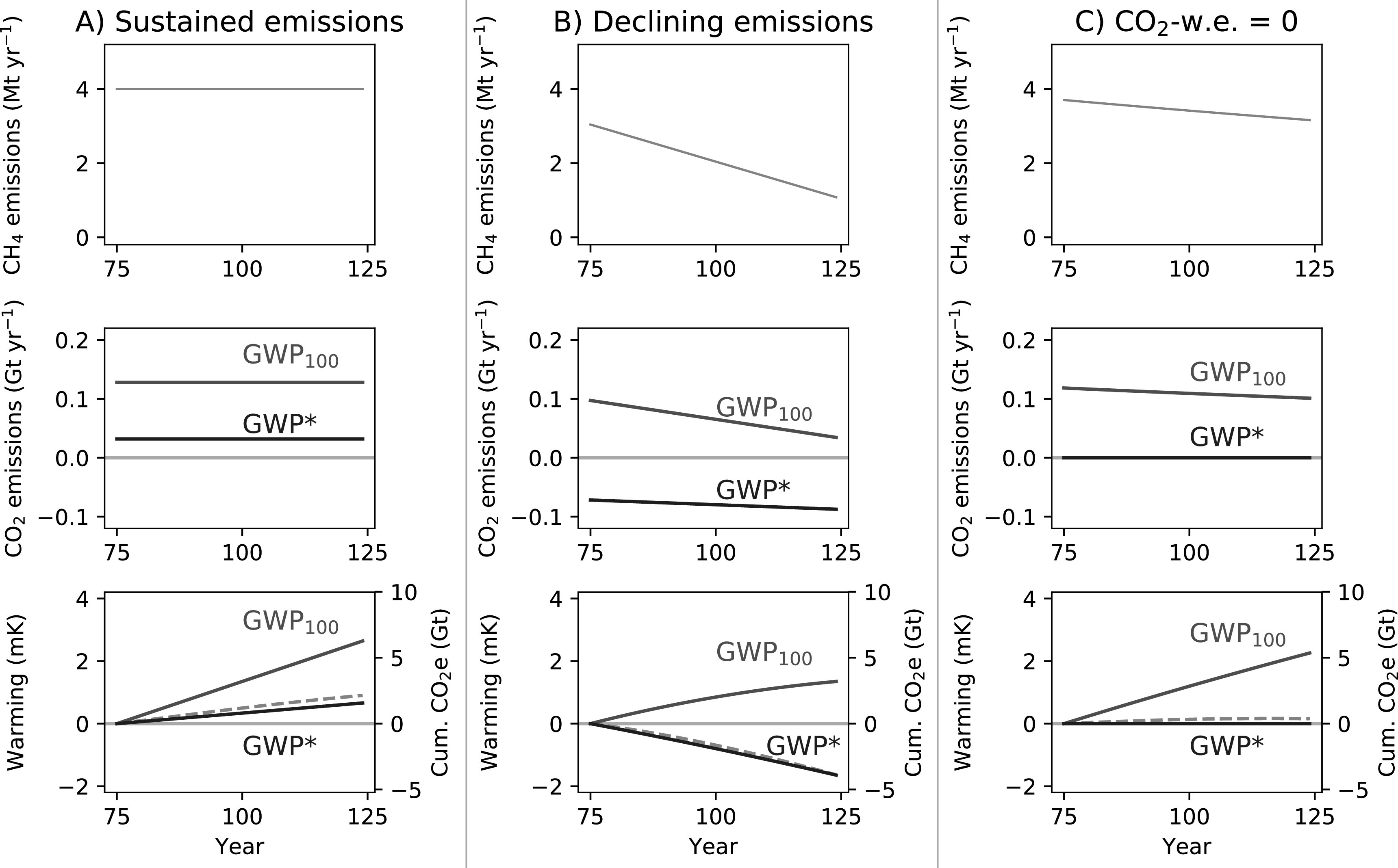
Demonstrating the behaviour of GWP* and GWP_100_ CO_2_-equivalents and warming resulting from methane scenarios with (A) sustained emissions, (B) linearly decreasing emissions rates and (C) a rate of decline such that the methane emissions are equivalent to 0 GWP* CO_2_-w.e. emissions. Figures show annual methane emissions (upper), annual CO_2_-equivalent emissions derived using both GWP_100_ (CO_2_-e, red line) and GWP* (CO_2_-w.e., blue line) (middle), and the relative temperature change resulting from the methane emissions (orange dashed line) overlaid with cumulative CO_2_-e (red line) and CO_2_-w.e. (blue line) emissions (lower).

We begin by considering scenarios with stable or declining methane emissions (figure 5). For ongoing stable methane emissions (figure 5(a)), the CO_2_-equivalent quantity is a constant annual emission under both GWP_100_ and GWP*, but GWP* CO_2_-w.e. is much less than GWP_100_ CO_2_-e (×0.25, following (2) with the suggested *s*), and cumulative CO_2_-w.e. (×TCRE) a much better match for the modelled warming. Consequently, if we sought to offset the continued warming from long-term stable methane emissions by annually sequestering an appropriate amount of CO_2_ derived using the GWP_100_ (i.e. a ‘net-zero emissions’ policy using GWP_100_), we would overstate the impacts of this methane and result in cooling, rather than temperature stabilisation [33, 34].

For declining methane emissions rates (figure 5(b)), the key point is as noted above: the falling temperatures under this scenario are equivalent to a removal of atmospheric CO_2_, as described using GWP* (unless the rate of decline in methane emissions is very gradual, see below). Under conventional use of GWP_100_, it is only possible to equate any ongoing methane emissions at all to a positive CO_2_ emission, suggesting continuing warming at odds with the real temperature response.

We can consider a special case where the decline in methane emissions rate is such that it is described by 0 GWP* CO_2_-w.e. emissions (figure 5(c)). This can be achieved through an annual decline in methane emissions of approximately 0.3% per year (for the *r* and *s* values suggested above, see supplementary material for derivation). As shown (figure 5(c)-lower), this scenario results in negligible additional warming despite ongoing, relatively high, methane emissions. Meanwhile, the large ongoing GWP_100_ CO_2_-e emissions would lead to significant continued temperature increases. A key point emerges which is obscured by conventional use of GWP_100_: to prevent further warming, it is necessary that net CO_2_ emissions are reduced to zero, but this is not the case for methane, where it is possible to have climatically sustainable ongoing emissions.

Any decline in methane emissions rate faster than the 0.3% p.a. decline noted above suggests cooling relative to the current temperature (figure 5(b), see [16] for discussion of the physical origin and uncertainty in this rate of decline). This is broadly the role of methane in ambitious mitigation pathways, where significant, permanent, reductions in methane emission rates can permit the emissions of a fixed amount of extra CO_2_, and hence the additional long-term warming it will cause, under a given temperature ceiling [35, 36]. This concept is explored further below when demonstrating the use of GWP* to assess alternative mitigations.

**Figure 6.**
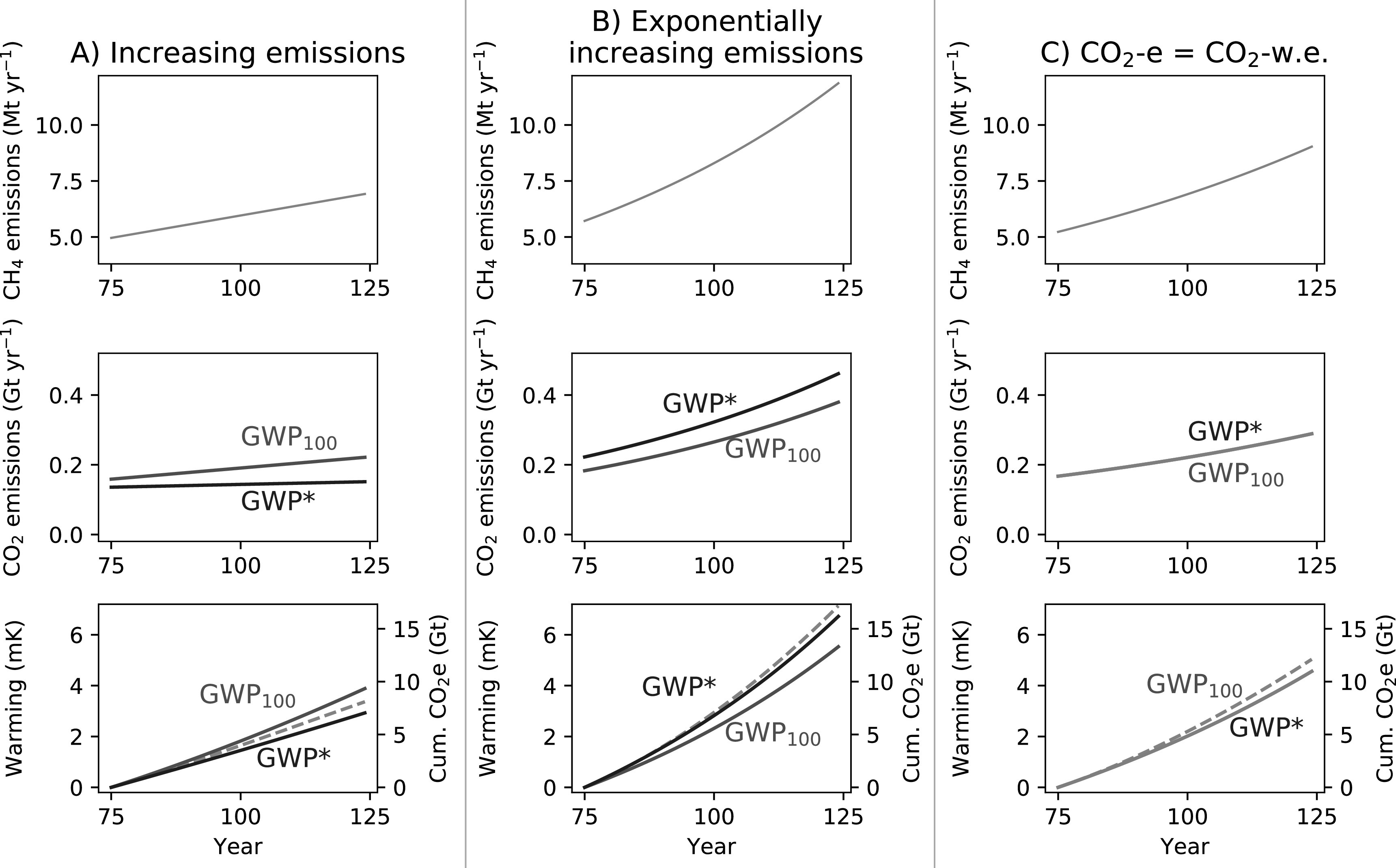
Demonstrating the behaviour of GWP* and GWP_100_ CO_2_-equivalents and warming resulting from methane scenarios with (A) a linear increase in emissions, (B) an exponentional increase in methane emissions emissions and (C) methane emissions increasing at a rate such that CO_2_-equivalents derived using either GWP* or GWP_100_ are equal. Figures show annual methane emissions (upper), annual CO_2_-equivalent emissions derived using both GWP_100_ (CO_2_-e, red line) and GWP* (CO_2_-w.e., blue line) (middle), and the relative temperature change resulting from the methane emissions (orange dashed line) overlaid with cumulative CO_2_-e (red line) and CO_2_-w.e. (blue line) emissions (lower).

It should be noted that these stable or declining temperatures are relative to present temperatures, as already experienced; not relative to before the emission was introduced. (i.e. for individual temperature subplots in figures 5 and 6, warming and cumulative CO_2_-equivalents are both from 0 in year 75, setting this as our reference point, but comparison with figure S4 (supplement) shows that this temperature is +5.3 mK relative to before the methane emission was introduced.) Long-term methane emitters therefore still have a warming legacy, and their continued emissions sustain elevated temperatures. It could thus be argued that, despite being able to achieve no additional warming through relatively small mitigations, continued methane emitters still have a responsibility to decrease emissions and mitigate climate change as much as possible, akin to holding countries responsible for their national contribution to observed global warming [37]. Where historical emissions are known, cumulative GWP* CO_2_-w.e. emissions to date can be used to indicate this responsibility, while annual GWP_100_ CO_2_-e, the basis of most policy targets, cannot.

But given that any CO_2_ emissions yield continued warming, by communicating emissions and designing strategies based primarily on reductions to ongoing annual CO_2_ emissions, there is an implicit baseline of no further CO_2_ emissions and hence no additional warming (particularly in relation to a stated ambition of net-zero CO_2_ emissions). Alternatively, if we considered an emissions-accounting framework that did reflect total warming contribution, and could describe responsibilities to reverse warming rather than just encourage no further temperature increases, the baseline for CO_2_ emitters would not just be their current or recent annual CO_2_ emission rates, but total cumulative CO_2_ emissions to date, and they would have a responsibility to sequester carbon and reverse historic contributions, rather than simply reduce ongoing emissions. GWP* could provide a means to account for different gases within such a framework, setting climatically meaningful responsibilities for diverse sectors producing distinct emissions. Meanwhile, using annual GWP_100_ CO_2_-emission rates, as is common at present, results in a lack of clarity on the different historical warming contributions between sectors or the distinct climate outcomes of reducing emissions of different gases.

#### 3.3.2. Increasing methane emissions

Next, we consider increases in methane emissions (figure 6; note the change in scales to expand the upper limits from figure 5). A linear increase in methane emissions (figure 6(a)) is represented by a relatively constant CO2-w.e. emission over time, as the change in rate of methane emissions $\tfrac{{\rm{\Delta }}{E}_{{\rm{SLCP}}}}{{\rm{\Delta }}t}$, is stable, which translates to a mostly linear increase in temperatures. Conventional use of GWP_100_, however, implies a linear increase in the rate of CO_2_-e emissions, corresponding to a more strongly exponential temperature increase.

(Note that for this period, the real temperature response lies approximately between the illustrated cumulative GWP* and GWP_100_ CO_2_-equivalents, but figure S5 in the supplementary materials below reveals that the more linear fit for GWP* CO_2_-w.e. is a much better match over the longer-term.)

In all of the 50 year scenarios described thus far, GWP_100_ CO_2_-e has been greater than GWP* CO_2_-w.e., but this is not a universal feature of GWP*. For an exponential increase in methane emissions, as shown in Fig 6, GWP* will appropriately describe the methane emissions as a greater CO_2_-equivalent quantity than GWP_100_ (see also the large initial GWP* CO_2_-w.e. for introducing a methane emission shown in figures 3 and 4).

Under both of these scenarios of increasing methane emissions the CO_2_-equivalent emissions from GWP_100_ are closer to those derived using GWP*, and consequently the actual warming from methane, than the stable and declining emissions scenarios. Another interesting case in this context is where methane emissions increase at a rate such that both GWP_100_ and GWP* CO_2_-equivalents are consistently equal (i.e. GWP CO_2_-w.e. = GWP_100_ CO_2_-e, figure 6(c)), which occurs under an exponential increase in methane emission rates of approximately 1% per year for the coefficient values suggested above (see supplementary material for derivation). If methane emissions increase at greater than 1% per year, GWP* CO_2_-w.e. will therefore be greater than GWP_100_ CO_2_-e, but vice versa if the increase is less than 1% per year.

### 3.4. Cumulative GWP* CO_2_-w.e. emissions and timing of emissions reductions

**Figure 7.**
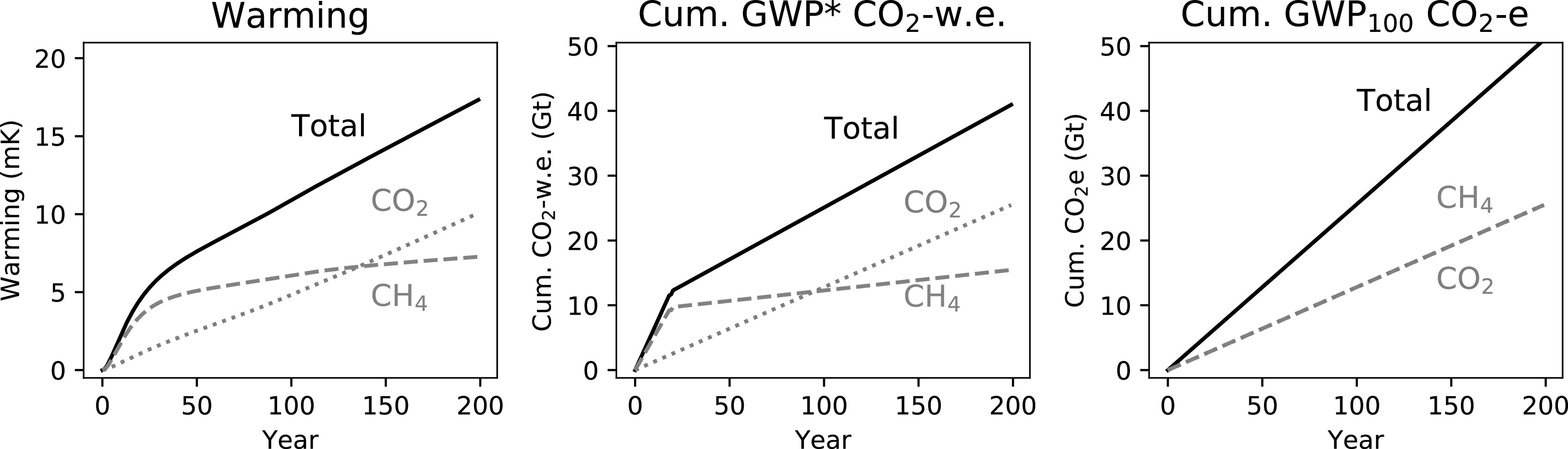
A demonstration of the warming resulting from introducing and then sustaining emissions of both methane and CO_2_ emissions, and the corresponding cumulative emissions using either GWP* or conventional application of GWP_100_.

Finally, we build on the principles and demonstrations above to provide a simple example of how GWP* can be used to better appraise GHG mitigation strategies. These mitigations are made to a baseline multi-gas scenario (figure 7), where emissions of both methane and CO_2_ are introduced and then maintained at the annual emission rates used in figure 1. This scenario leads to a rapid initial increase in warming, primarily from the methane, followed by a slower temperature increase driven predominantly from the consistent rate of CO_2_-induced warming. Summing the two sets of cumulative CO_2_-w.e. emissions provides an approximation of this, while the two sets of cumulative CO_2_e emissions suggest they have the same linear impacts, and so the total is simply a greater rate of linear increase.

**Figure 8.**
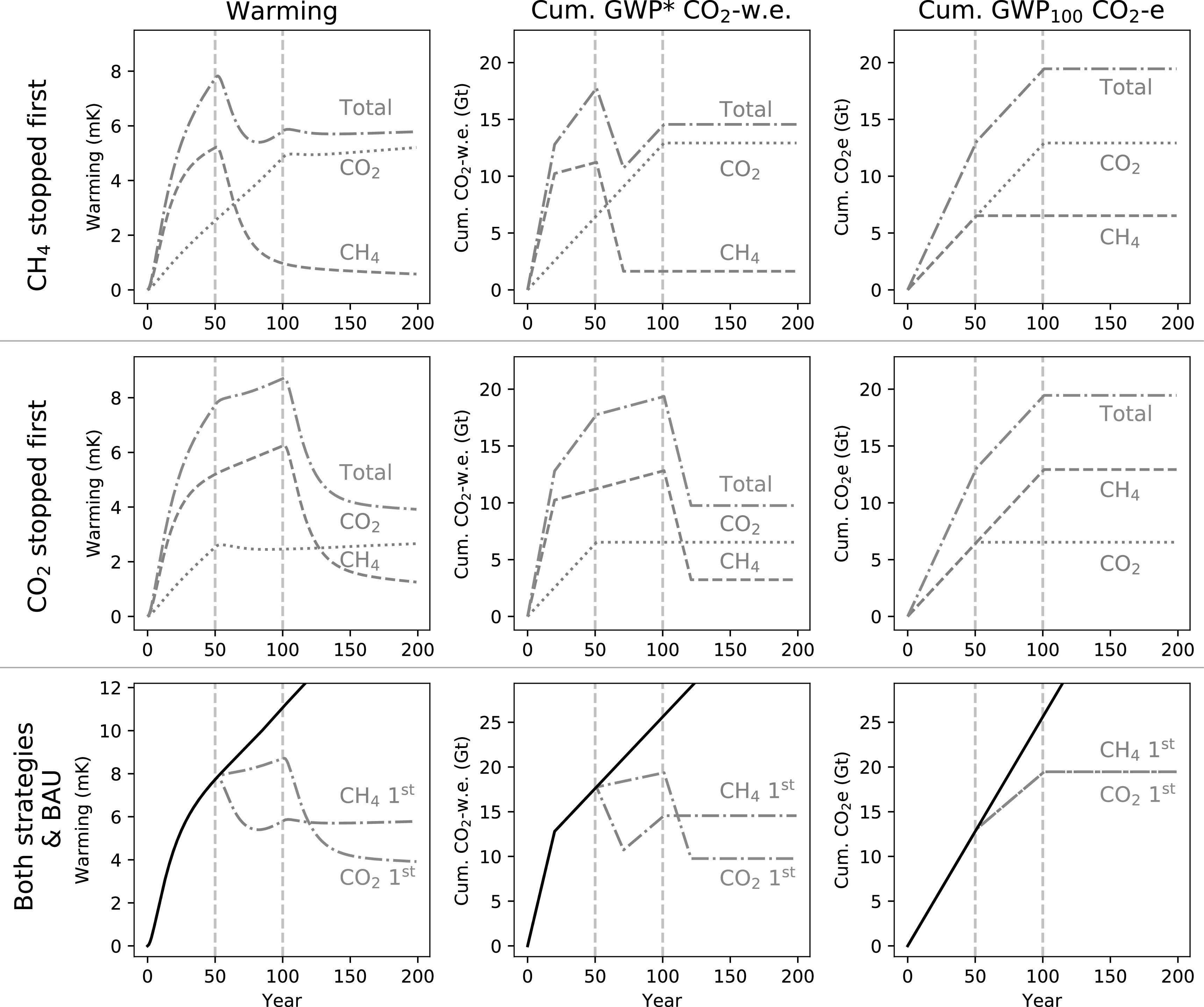
Demonstration of a scenario introducing and maintaining CH_4_ and CO_2_ emissions, then stopping emissions of one gas after 50 years and the other after 100 years. The first row demonstrates a strategy where CH_4_ is stopped first and CO_2_ second; the second row where CO_2_ is stopped first. The third row shows both of these mitigation pathways, in addition to the ‘Business as Usual’ (black line) constant emissions scenario from figure 7. Dotted lines illustrate the warming impact or cumulative CO_2_-equivalent emissions for CO_2_ only, dashed lines for methane online, and dot-dash lines for the total impacts/cumulative emissions of both gases together.

We can demonstrate the utility of multi-gas cumulative CO_2_-w.e. totals in a decision making context by considering how they would describe alternative mitigation pathways, as in figure 8. In this scenario, the emissions of one gas cease in year 50, and then the emissions of the remaining gas in year 100. Stopping methane first results in a large initial reversal of recent warming, but temperatures then start to rise again due to the ongoing CO_2_ emissions. Temperature then stabilises at the temperature reached in year 100 when CO_2_ emissions are also stopped. Stopping CO_2_ first, we see that the rate of warming declines, and then when methane emissions stop in year 100 we have a significant reversal of warming, stabilising at a lower long-term temperature than in the methane-first scenario. Cumulative CO_2_-w.e. provides a clear indication of these dynamics, while cumulative CO_2_e suggests either strategy would lead to the same response, but which represents neither scenario.

These two strategies illustrate an important principle: that focussing on methane at the expense of (a GWP_100_ equivalent quantity of) CO_2_ is not simply a prioritisation of short- over long-term impacts, it is a trade-off. There is a large initial benefit (indeed, a significant reversal of total warming, in this scenario) from focussing on methane, but the more that action on CO_2_ is postponed, the higher the temperature that we are locked in to. Consequently, reducing methane emissions, though beneficial, does not ‘buy time’ for delayed CO_2_ mitigation in the sense that it provides an equivalent pathway to acting on CO_2_. It is universally better to stop CO_2_ emissions early, as the sooner they are stopped, the sooner they stop compounding and the less CO_2_-induced warming we are committed to for the very long-term [38]. Rather, the negative CO_2_-w.e. generated by *permanently reducing methane emission rates* expands the allowable space for emissions of cumulative GHGs within a total [CO_2_-w.e.] emissions budget, and hence permits a greater *one-off fixed quantity* of CO_2_ emissions under a given temperature ceiling. The inverse implication is that the longer we fail to decarbonise, and hence add to the cumulative stock of CO_2_ emitted, then the smaller the permissible rate of ongoing, sustainable methane emissions within a given emissions budget (and hence also a given temperature ceiling). In this context, it is worth noting that annual fossil fuel CO_2_ emissions were the highest ever in 2019 and predicted to continue increasing in 2020 [39].

**Figure 9.**
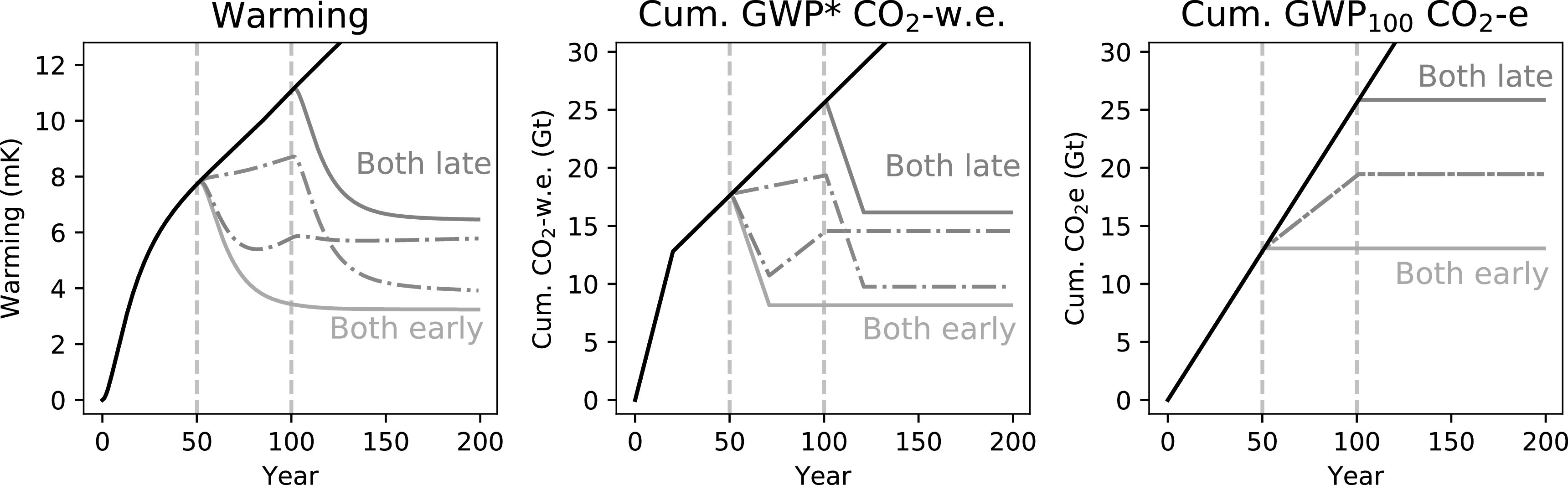
Demonstration of a scenario of constant CH_4_ and CO_2_ emissions, stopping one gas at 50 years and the other at 100 years, as in figure 8 above, or stopping ‘both early’ (both at 50 years) or ‘both late’ (both at 100 years).

We can complete the range of scenarios in this framework by adding the cases where we stop emissions of both gases earlier or later figure 9. As should be self-evident, the climatically optimum strategy is to reduce emissions of any greenhouse gases as early as possible. Stopping both gases early results in rapid cooling from the cessation of methane emissions, and also prevents the ongoing temperature increases that would occur from sustained CO_2_ emissions. The scenario in which both emissions are stopped later highlights a further implication of the principles described above: while the point at which methane emissions are stopped can have an important impact on peak warming (so long as CO_2_ are also stopped contemporaneously or shortly afterwards), as most of the warming impact of methane depends on the rate of emissions, it is largely time-independent. Delaying action on methane does not have as significant an impact on long-term temperature as delaying action on CO_2_ emissions; the eventual temperature we remain committed to, once both emissions are stopped, is predominantly determined by the timing of CO_2_ mitigation.

The ‘stock’ component, respresenting the long-term temperature adjustment to methane emissions, is still important, and the scale of contemporary methane emissions is such that this element could equate to a significant increase in total allowable CO_2_ emissions for warming of 1.5° C if action is taken on methane earlier rather than later [35], but by a much smaller amount than the direct GWP_100_ equivalence between methane and CO_2_ would suggest.

## 4. Concluding remarks

CO_2_-equivalents have become a near-universal means of reporting greenhouse gas emissions, and in many cases are used to directly infer their climate impacts or role in mitigation strategies—even if such an expansive application was never intended. Given this, it is important to have a means of deriving CO_2_-equivalents that provides a reliable link between reported emissions and their warming impacts. As demonstrated, in many cases conventional use of GWP_100_ does not achieve this, while GWP* does.

Using GWP_100_ to direct climate change mitigation strategy could be unfair, inefficient, and dangerous. Unfair, as it does not provide a clear link between emissions and climate change contribution, and could lead to an expectation that some actors (long-term methane emitters) have to undo their past warming, while others (CO_2_ emitters) merely have to limit further temperature increases. Inefficient, as it would overstate the level of action needed to offset long-term sustained methane emissions, while simultaneously undervaluing the potential short-term benefits of reducing these methane emissions. Dangerous, as it can greatly understate the impacts of increasing methane emissions, and obscure the fundamental need for net-zero CO_2_ emissions as soon as possible, regardless of what mitigations are made to shorter-lived climate pollutants.

There is an additional danger, which is to the perceived environmental integrity of climate policy. Basing climate policies and emission trading systems on a metric that demonstrably fails to reflect the impact of different emissions on global temperature, while at the same time claiming these are designed to deliver a long-term temperature goal, risks undermining confidence in the entire strategy. GWP* provides a straightforward means of dealing with these issues, calculating genuinely warming-equivalent emissions using information that is already being reported in the UNFCCC system.

## Data availability statement

Any data that support the findings of this study are included within the article. The modelling and background emissions data used are as described in [20], and can be found at https://github.com/OMS-NetZero/FAIR.

## References

[1] Myhre G (2013). Anthropogenic and Natural Radiative Forcing.

[2] UNFCCC (2018). Presidency consultations on modalities, procedures and guidelines under the Paris Agreement with a focus on transparency. Draft Report Version 1.

[3] ISO 14044 (2006). Environmental Management—Life Cycle Assessment—Requirements and Guidelines.

[4] Smith S J, Wigley M L (2000). Global warming potentials: I. Climatic implications of emissions reductions. Clim. Change.

[5] Fuglestvedt J S, Berntsen T K, Godal O, Sausen R, Shine K P, Skodvin T (2003). Metrics of climate change: assessing radiative forcing and emission indices. Clim. Change.

[6] Shine K P (2009). The global warming potentialhe need for an interdisciplinary retrial. Clim. Change.

[7] Pierrehumbert R T (2014). Short-lived climate pollution. Annu. Rev. Earth Planet. Sci..

[8] Archer D, Brovkin V (2008). The millennial atmospheric lifetime of anthropogenic CO_2_. Clim. Change.

[9] Eby M, Zickfeld K, Montenegro A, Archer D, Meissner K J, Weaver A J (2009). Lifetime of anthropogenic climate change: millennial time scales of potential CO_2_ and surface temperature perturbations. J. Clim..

[10] Knutti R, Rogelj J (2015). The legacy of our CO_2_ emissions: a clash of scientific facts, politics and ethics. Clim. Change.

[11] Smith S M, Lowe J A, Bowerman N H A, Gohar L K, Huntingford C, Allen M R (2012). Equivalence of greenhouse-gas emissions for peak temperature limits. Nat. Clim. Change.

[12] Lauder A R, Enting I G, Carter J O, Clisby N, Cowie A L, Henry B K, Raupach M R (2013). Offsetting methane emissions an alternative to emission equivalence metrics. Int. J. Greenhouse Gas Control.

[13] Pierrehumbert R T, Eshel G (2015). Climate impact of beef: an analysis considering multiple time scales and production methods without use of global warming potentials. Environ. Res. Lett..

[14] Collins W J, Frame D J, Fuglestvedt J, Shine K P (2019). Stable climate metrics for emissions of short and long-lived species—combining steps and pulses. Environ. Res. Lett..

[15] Allen M R (2016). New use of global warming potentials to compare cumulative and short-lived climate pollutants. Nat. Clim. Change.

[16] Cain M, Lynch J, Allen M R, Fuglestvedt J S, Macey A H, Frame D J (2019). Improved calculation of warming-equivalent emissions for short-lived climate pollutants. NPJ Clim. Atmos. Sci..

[17] Allen M R, Shine K P, Fuglestvedt J S, Millar R J, Cain M, Frame D J, Macey A H (2018). A solution to the misrepresentations of CO_2_-equivalent emissions of short-lived climate pollutants under ambitious mitigation. NPJ Clim. Atmos. Sci..

[18] Solomon S, Daniel J S, Sanford T J, Murphy D M, Plattner G-K, Knutti R, Friedlingstein P (2010). Persistence of climate changes due to a range of greenhouse gases. Proc. Natl Acad. Sci..

[19] Goodwin P, Williams R G, Ridgwell A (2015). Sensitivity of climate to cumulative carbon emissions due to compensation of ocean heat and carbon uptake. Nat. Geosci..

[20] Smith C J, Forster P M, Allen M, Leach N, Millar R J, Passerello G A, Regayre L A (2018). Fair v1.3: a simple emissions-based impulse response and carbon cycle model. Geosci. Model Dev..

[21] Etminan M, Myhre G, Highwood E J, Shine K P (2016). Radiative forcing of carbon dioxide, methane, and nitrous oxide: a significant revision of the methane radiative forcing. Geophys. Res. Lett..

[22] Meinshausen M (2011). The rcp greenhouse gas concentrations and their extensions from 1765 to 2300. Clim. Change.

[23] Brown P (2018). UK Greenhouse Gas Inventory, 1990 to 2016. Annual Report for Submission under the Framework Convention on Climate Change. Ricardo-AEA Report ED62689/0/CD8977/PB.

[24] Janssens-Maenhout G (2017). Edgar v4.3.2 global atlas of the three major greenhouse gas emissions for the period 1970–2012. Earth Syst. Sci. Data Discuss.

[25] Jenkins S, Millar R J, Leach N, Allen M R (2018). Framing climate goals in terms of cumulative CO_2_-forcing-equivalent emissions. Geophys. Res. Lett..

[26] Collins M (2013). Long-Term Climate Change: Projections, Commitments and Irreversibility.

[27] Damon Matthews H, Zickfeld K, Knutti R, Allen M R (2018). Focus on cumulative emissions, global carbon budgets and the implications for climate mitigation targets. Environ. Res. Lett..

[28] Reisinger A, Meinshausen M, Manning M (2011). Future changes in global warming potentials under representative concentration pathways. Environ. Res. Lett..

[29] Shine K P, Fuglestvedt J S, Hailemariam K, Stuber N (2005). Alternatives to the global warming potential for comparing climate impacts of emissions of greenhouse gases. Clim. Change.

[30] Peters G P, Aamaas B, Berntsen T, Fuglestvedt J S (2011). The integrated global temperature change potential (iGTP) and relationships between emission metrics. Environ. Res. Lett..

[31] Azar C, Johansson D J A (2012). On the relationship between metrics to compare greenhouse gases—the case of igtp gwp and sgtp. Earth Syst. Dyn..

[32] Allen M, Cain M, Shine K P (2017). Climate Metrics under Ambitious Mitigation. Oxford Martin School Briefing.

[33] Fuglestvedt J, Rogelj J, Millar R J, Allen M, Boucher O, Cain M, Forster P M, Kriegler E, Shindell D (2018). Implications of possible interpretations of ‘greenhouse gas balance’ in the paris agreement. Phil. Trans. R. Soc. A.

[34] Tanaka K, O’Neill B C (2018). The paris agreement zero-emissions goal is not always consistent with the 1.5° C and 2° C temperature targets. Nat. Clim. Change.

[35] Collins W J (2018). Increased importance of methane reduction for a 1.5 degree target. Environ. Res. Lett..

[36] Rogelj J (2018). Scenarios towards limiting global mean temperature increase below 1.5° C. Nat. Clim. Change.

[37] Matthews H D, Graham T L, Keverian S, Lamontagne C, Seto D, Smith T J (2014). National contributions to observed global warming. Environ. Res. Lett..

[38] Bowerman N H A, Frame D J, Huntingford C, Lowe J A, Smith S M, Allen M R (2013). The role of short-lived climate pollutants in meeting temperature goals. Nat. Clim. Change.

[39] Jackson R B, Friedlingstein P, Andrew R M, Canadell J G, Le Quéré C, Peters G P (2019). Persistent fossil fuel growth threatens the paris agreement and planetary health. Environ. Res. Lett..

