# Supplementary material for: Demonstrating GWP*: a means of reporting warming-equivalent emissions that captures the contrasting impacts of short- and longlived climate pollutants
Source: Environ Res Lett. Author manuscript; Available in PMC 2020 May 11. (PMC7212016; doi:10.1088/1748-9326/ab6d7e)
Supplement: Supplementary file 1 [file EMS86266-supplement-Supplement.pdf]

Supplement to: Demonstrating GWP*

John Lynch[1], Michelle Cain[1], Raymond Pierrehumbert[1], Myles Allen[1,2]

[1]Department of Physics, University of Oxford
[2]Environmental Change Institute, University of Oxford

Contents

1 Transient Climate Response to cumulative carbon Emissions (TCRE) 2

2 CO_2 equivalence of methane emissions under alternative metrics to the 100-year Global Warming Potential 4

3 Design of the 50-year illustrative scenarios 7

4 Derivations of illustrative 'special cases' for GWP* 9

5 Longer-term dynamics of linear increases in methane emission rate 11

1 Transient Climate Response to cumulative carbon Emissions (TCRE)

The Transient Climate Response to cumulative carbon Emissions, or TCRE, has become an important concept in climate science over the past decade [1, 2, 3], and forms a key component of recent IPCC communications, providing the context for 'carbon budgets' and their significance for mitigation pathways [4, 5]. Providing a form of 'carbon dioxide equivalence' for short-lived greenhouse gases that is compatible with carbon budgets is key to both the purpose and construction of GWP* [6, 7].

As described in the main text, as the relationship between cumulative CO_2 emissions and total warming is approximately linear, the gradient of this line, the TCRE, can be used to link temperatures to emissions. Many of the figures in the paper suggest this correspondence by overlaying cumulative CO_2-equivalents describing methane emissions with the temperature impact of these methane emissions. Here we describe the TCRE used in this study.

A number of different models and approaches have been used to estimate the TCRE, producing a range of values (see [3] for a recent review), and has been shown to have some time and scenario dependence [8]. In this letter we used our modelling framework to derive an appropriate TCRE in order to ensure it was internally consistent with the scenarios and outcomes shown here. To achieve this, we used the RCP4.5 baseline scenario to model warming, then estimated the CO_2-induced warming at a given time (ΔT_{CO_2}) using a simple approach following [9]:

$$\Delta T_{\mathrm{CO_2}} = \Delta T \times \frac{RF_{\mathrm{CO_2}}}{RF_{all}} \qquad \text{(Eqn. S1)}$$

where ΔT is the total warming, and RF_{all} and $RF_{\mathrm{CO_2}}$ the total and CO_2-only radiative forcings at that time, respectively. The TCRE was then derived using linear regression to determine the fit between cumulative carbon emissions and warming. Both cumulative CO_2 emissions are warming are relative to 1870 when reliable data begins [10, 11], and the regression covers the period from this date to 2200, the latest year included in any of our scenarios. The intercept was constrained to 0 to establish a meaningful and consistent framework for cumulative emissions to indicate further temperature change.

As shown in Fig S1, FaIR returns a remarkably linear relationship between cumulative emissions warming for RCP4.5 from 1870 to 2200. The linear regression therefore gives a very strong fit ($p < 0.001, R^2 = 0.99$), suggesting a gradient (i.e. the TCRE) of 1.524 K per Tt C, or, 0.42 K per Gt CO_2, used for all figures in the paper combining both temperature and cumulative CO_2-equivalent emissions. This TCRE value is within the IPCC's likely range of 0.8 to 2.5 K per Tt C [4], and between the median

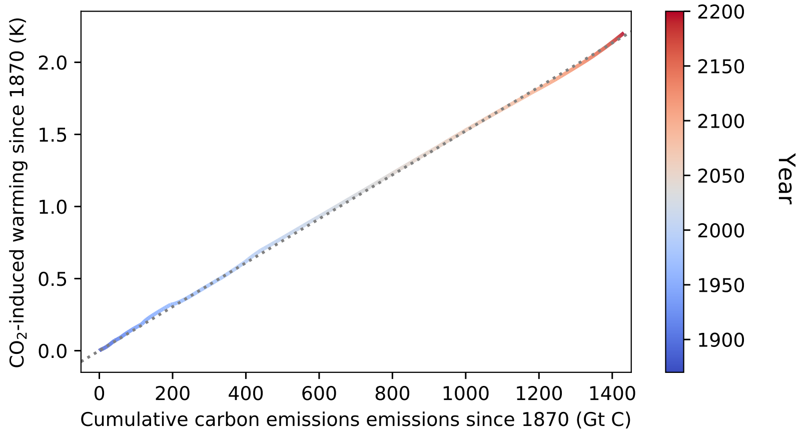

Figure S1: Cumulative carbon emissions from the RCP4.5 pathway and their resultant warming for 1870 to 2200 (multi-coloured solid line), and a linear regression to demonstrate this relationship and hence the Transient Climate Response to cumulative carbon Emissions (TCRE, dotted grey line – gradient of 1.52 K per Tt C).

(1.6) and observation constrained best-estimate (1.35) from a recent review of full-complexity climate models [12].

Under other scenarios the TCRE may change, with some models suggesting a slightly concave relationship whereby the amount of warming per unit of CO_2 emitted declines at high cumulative CO_2 emissions [13], which would reduce the accuracy of a direct correspondence between emissions and warming inferred here. However, so long as the GWP is updated to maintain the correspondence between in radiative forcing resulting from emissions of different gases (e.g. incorporating relative changes in atmospheric lifespan and radiative efficiency anticipated as atmospheric conditions change as a result of future emissions trajectories [14]), any approach used to relate CO_2 emissions to temperature change (for example, an alternative or non-linear TCRE) should also be applicable to short-lived greenhouse gases described as GWP* CO_2-warming equivalents. Confirming and exploring these relationships for a range of emissions scenarios and climate models may be a useful avenue for further research.

2 CO_2 equivalence of methane emissions under alternative metrics to the 100-year Global Warming Potential

Alternative static CO_2-equivalence metrics to the 100-year Global Warming Potential will have the same general issues identidied in the main text, as the fundamental problem is that individual emissions of methane and CO_2 are simply not directy equivalent, and attempts to treat them as if they are will always fail to reflect their different dynamic impacts. We illustrate this here by adding the CO_2 equivalent emissions as defined using the 20-year Global Warming Potential (GWP_{20}) and the 100-year Global Temperature change Potential (GTP_{100}) to our demonstration of introduced and then sustained methane emissions (Fig. S2, expanding on Fig. 3 in the main text). These metrics were used as they are the most common alternatives to GWP_{100}, and have been recommended as complementary reporting options to GWP_{100} in order to reflect the very near-term (GWP_{20}) and longer-term (GTP_{100}) impacts of emissions [15].

While GWP_{20} comes closer to representing the scale of the initial impacts of introducing a new source of methane emissions (though still undervaluing them relative to GWP*), if these emissions continue at a stable rate, it greatly overstates the warming contribution of methane emissions. Meanwhile, GTP_{100} is nearer to the relatively small additional warming expected from long-term sustained methane emissions, but as it does not capture the strong effects of changing methane emission rates, understates the overall warming. The fundamental problem of being able to capture the automatic reversibility of non-cumulative pollutants (i.e. methane) is again highlighted in Fig. S3, expanding on Fig. 4 in the main text)

It should be noted that a discrepency is introduced in this supplementary figure, as while the modelling and both GWP* and GWP_{100} are reported use the latest research reflecting an increased methane forcing [16], GWP_{20} and GTP_{100} use older values directly from [17]. The GWP_{20} and GTP_{100} values for methane will also likely increase in the next IPCC Assessment Report in light of this research. However, if the upwards revisions of methane's GWP_{20} and GTP_{100} are similar to the relative change in GWP_{100}, as would be expected, the differences will be relatively small and the overall picture will be as described above.

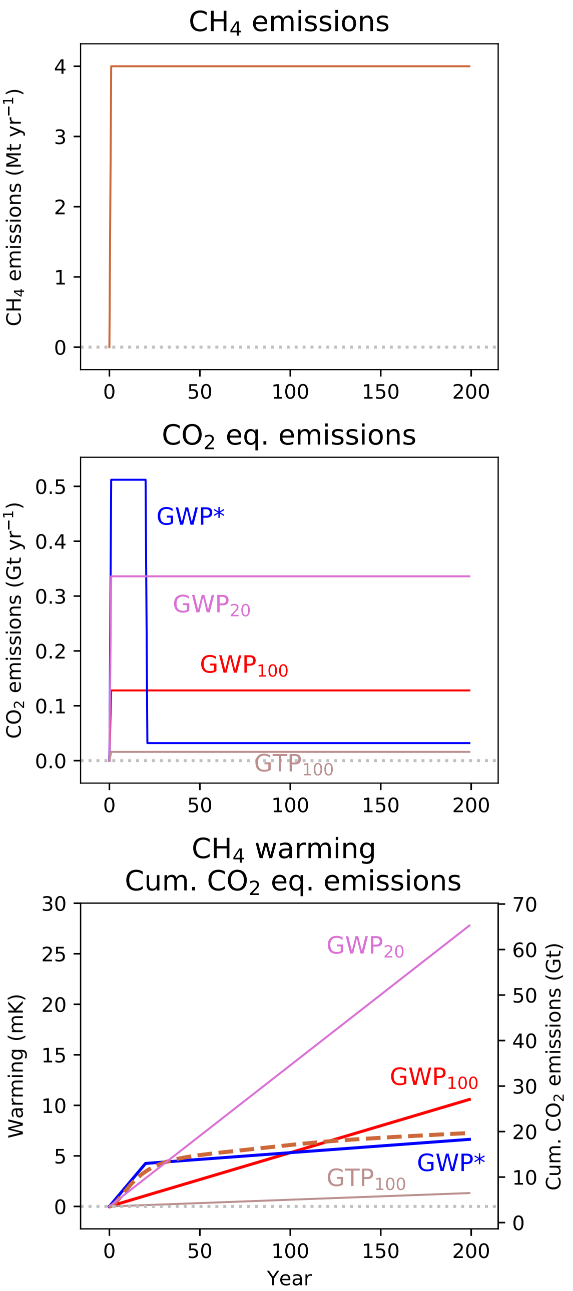

Figure S2: A demonstration of a step-change to sustained CH_4 emissions (as in Figure 3 in main text), and corresponding annual CO_2-equivalent emissions and the warming resulting from those CH_4 emissions overlaid with cumulative GWP*, GWP100, GWP20 and GTP100 CO_2-equivalent emissions.

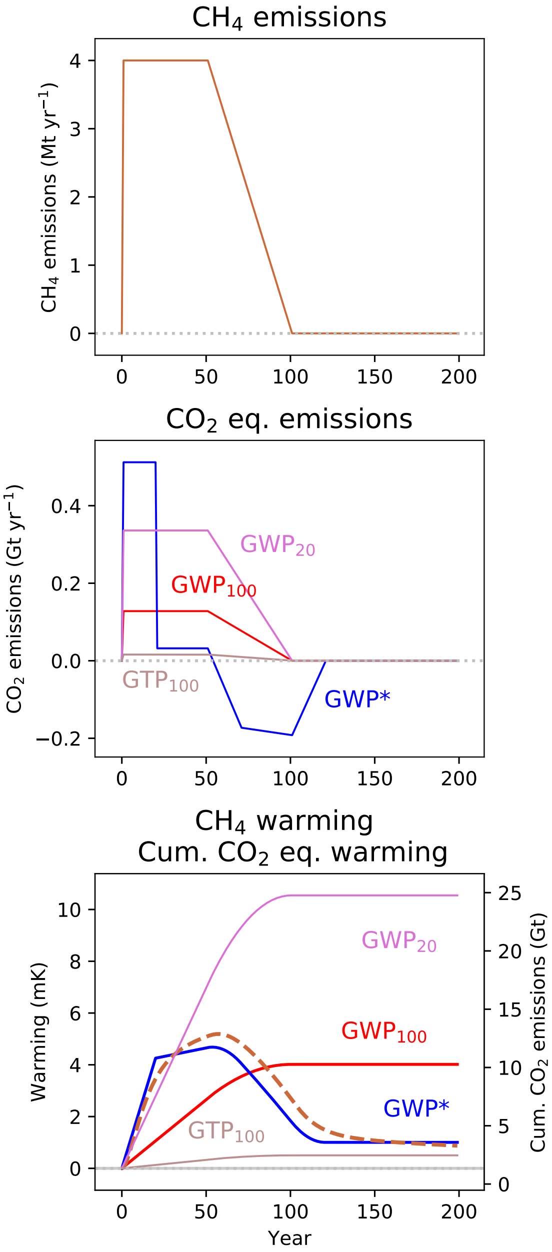

Figure S3: A demonstration of a step-change to sustained CH_4 emissions for 50 years followed by a decline to 0 emissions over the following 50 years (as in Figure 4 in main text), and corresponding annual CO_2-equivalent emissions and the warming resulting from those CH_4 emissions overlaid with cumulative GWP*, GWP100, GWP20 and GTP100 CO_2-equivalent emissions.

3 Design of the 50-year illustrative scenarios

As stated in the main text, to set up scenarios that provide simple illustrations of how changes in methane emission rates are described as GWP* and GWP_{100} CO_2-equivalents, it is useful (and more policy-relevant) to begin from a position of some ongoing methane emissions.

To achieve this, all of our illustrative scenarios follow an initial 50 years of a sustained methane emission, as described for Fig. 1 in the main text. Following this, we switch to the methane emissions pathway of interest, and derive the two sets of CO_2-equivalent emissions 25 years into this new trajectory so that the GWP* emissions rate is stable and thus capturing only the key dynamics being illustrated, rather than the transition from stable emissions to the new rates. The concept is demonstrated in Fig. S4, showing how, in this demonstration of falling methane emission rates (Fig. S4-upper), we start with the same step-change to sustained emissions for 50 years, at which point we change to declining emission rates. After 75 years (25 years after the change in emissions pathway), the CO_2-equivalents are calculated, and cumulative CO_2-equivalents. plotted alongside the warming resulting from the methane emissions (Fig. S4-lower). This demonstration corresponds to Fig 5b in the main text, where only the core 50-year window is shown, and the warming changed from a baseline set at year 75 instead of year 0 for the lower row of fig5, to illustrate the use of CO_2' equivalents to anticipate temperature change relative to the year that these equivalents are calculated (discussed further in the main text).

The generic modelling framework employed here means that this 50-year period represents 2075-2125, which likely remains a useful timeframe to consider the impacts of different emissions pathways, but we also remind the reader that these models are to demonstrate broad principles, and similar dynamics would also emerge for contemporary changes to currently ongoing methane emissions. Similarly, our basic starting point of 50-years of stable methane emissions is chosen for simplicity, but the concepts would also apply to more complex, realistic pathways.

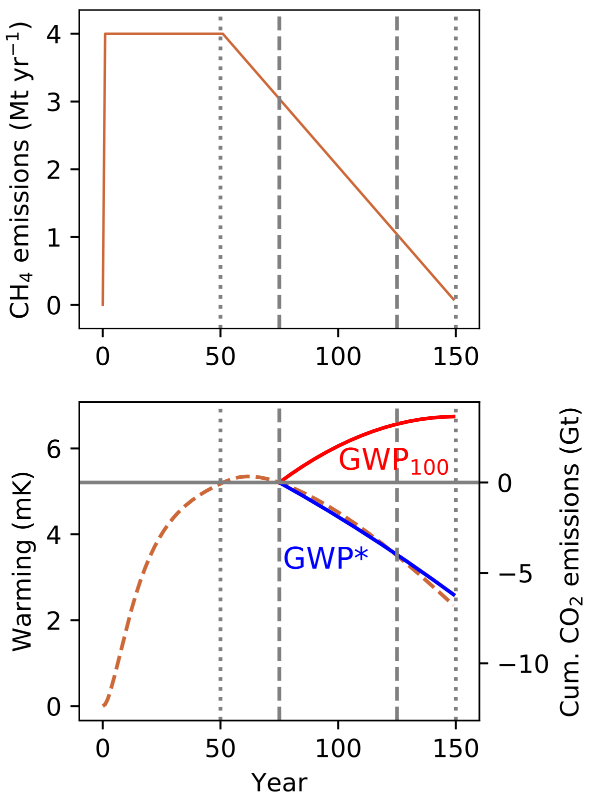

Figure S4: Overview of how the methane emissions scenarios (upper) as used for Figs.5 and 6 (here, showing 5b) in the main paper are defined such that there is an initial period of 50 years before changing to an illustrative new emissions pathway (here steadily declining methane emissions) that continues for 100 years, with the central 50 year component of this (years 75-125) used to generate cumulative GWP_{100} and GWP* CO_2-equivalent emissions for comparison with the warming from methane (lower).

4 Derivations of illustrative 'special cases' for GWP*

The GWP* equation (Eqn. 2 in main text, reiterated as (Eqn. S2) below)

$$E_{\mathrm{CO}_2-w.e.(SLCP)} = (r \times \frac{\Delta E_{SLCP}}{\Delta t} \times H + s \times E_{SLCP}) \times \mathrm{GWP}_H \quad \text{(Eqn. S2)}$$

can be rearranged to return a number special cases that illustrate key dynamics, as shown in the main paper.

Using GWP*, ongoing methane emissions can be equivalent to 0 CO_2 emissions, i.e. indicating no additional warming, where

$$r \times \frac{\Delta E_{SLCP}}{\Delta t} \times H + s \times E_{SLCP} = 0 \quad \text{(Eqn. S3)}$$

This could be considered as the rate of decline in methane emissions required to offset the on-going warming that results from the slow component of the climate response (or the change required such that the 'rate' component can offset the 'stock' component, in terms of the GWP* equation). Substituting ΔE for the emissions at two specific time-points, i.e. $\Delta E = E_{(t+\Delta t)} - E_t$, and rerranging, the required change in emissions between two given years is:

$$E_{(t+\Delta t)} = \frac{E_t}{(1 + \frac{s\Delta t}{rH})} \quad \text{(Eqn. S4)}$$

i.e the rate of emissions must fall by a proportion of $\frac{1}{(1+\frac{s\Delta t}{rH})}$ every Δt years. This reduction across the Δt period can also be spread out in a manner that consistently returns 0 GWP* CO_2-w.e. for each individual year:

$$E_{(t+1)} = E_t \times \sqrt[\Delta t]{\frac{1}{(1 + \frac{s\Delta t}{rH})}} \quad \text{(Eqn. S5)}$$

As stated in the main text, when $\Delta t = 20$, $r = 0.75$ and $s = 0.25$, this gives an annual rate of decline in short-lived GHG emission rates of 0.322% .

Another special instance occurs when the CO_2-equivalents derived using GWP_{100} and GWP* are equal. This is the case when

$$r \times \frac{\Delta E_{SLCP}}{\Delta t} \times H + s \times E_{SLCP} = E_{SLCP} \quad \text{(Eqn. S6)}$$

which can be rearranged to give:

$$\frac{\Delta E_{SLCP}}{\Delta t} = \frac{(1-s)}{rH} E_{SLCP} \quad \text{(Eqn. S7)}$$

If the left-hand-side of (Eqn. S7) is greater, then using GWP* will report a larger CO_2-equivalent emission than if using GWP_{100}, while if the right-hand side is greater, than using GWP_{100} will return the larger CO_2-equivalent emission.

Performing a similar substitution as in (Eqn. S4), and again spreading this out so that this is achieved consistently each year as in (Eqn. S5), we can describe a condition where emissions are maintained at this equivalence point:

$$E_{(t+1)} = E_t \times \sqrt[\Delta t]{\frac{1}{(1 - \frac{\Delta t(1-s)}{rH})}} \qquad \text{(Eqn. S8)}$$

In this case, GWP* and GWP_{100} consistently return the same CO_2-equivalent emissions (i.e. GWP* CO_2-w.e. = GWP_{100} CO_2-e), as shown in Fig. 7c. For $\Delta t = 20$, $r = 0.75$ and $s = 0.25$, this occurs when there is a continued increase in in short-lived GHG emission rates of 1.12% per year.

5 Longer-term dynamics of linear increases in methane emission rate

As noted in the main text, the warming resulting from a linear increase in methane emissions appeared approximately between both cumulative GWP_{100} CO_2-e and GWP* CO_2-w.e. for the 50 year window shown (Fig. 6a). Extending this scenario further (Fig. S5), it is revealed that the predominantly relatively linear warming that would be anticipated by cumulative GWP* CO_2-w.e. is much closer to the real methane warming profile than the path suggested by GWP_{100} CO_2-e.

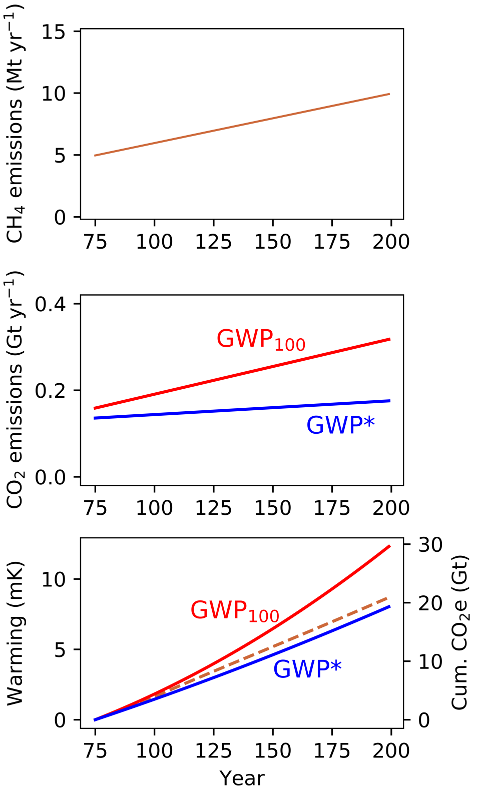

Figure S5: A longer-term view of the scenario shown in Fig. 6a. Methane emissions increase at a linear rate (upper), expressed as the two sets of CO_2-equivalents (middle). The lower panel then illustrates how cumulative CO_2-equivalents (solid lines, right y-axis) can then be used to describe modelled methane temperature change (dashed orange line, left y-axis).

References

[1] Myles R. Allen, David J. Frame, Chris Huntingford, Chris D. Jones, Jason A. Lowe, Malte Meinshausen, and Nicolai Meinshausen. Warming caused by cumulative carbon emissions towards the trillionth tonne. *Nature*, 458:1163, 2009.

[2] H. Damon Matthews, Nathan P. Gillett, Peter A. Stott, and Kirsten Zickfeld. The proportionality of global warming to cumulative carbon emissions. *Nature*, 459:829, 2009.

[3] H. Damon Matthews, Kirsten Zickfeld, Reto Knutti, and Myles R. Allen. Focus on cumulative emissions, global carbon budgets and the implications for climate mitigation targets. *Environmental Research Letters*, 13(1):010201, 2018.

[4] M. Collins, Reto Knutti, J. Arblaster, J.-L. Dufresne, T. Fichefet, Pierre Friedlingstein, X. Gao, W.J. Gutowski, T. Johns, G. Krinner, M. Shongwe, C. Tebaldi, A. J. Weaver, and M. Wehner. *Long-term Climate Change: Projections, Commitments and Irreversibility.* Cambridge University Press, Cambridge, United Kingdom and New York, NY, USA, 2013.

[5] IPCC. *Summary for Policymakers*, page 32. World Meteorological Organization, Genva, Switzerland, 2018.

[6] Myles R. Allen, Keith P. Shine, Jan S. Fuglestvedt, Richard J. Millar, Michelle Cain, David J. Frame, and Adrian H. Macey. A solution to the misrepresentations of co2-equivalent emissions of short-lived climate pollutants under ambitious mitigation. *npj Climate and Atmospheric Science*, 1(1):16, 2018.

[7] Michelle Cain, John Lynch, Myles R. Allen, Jan S. Fuglestvedt, Adrian H. Macey, and David J. Frame. Improved calculation of warming-equivalent emissions for short-lived climate pollutants. *Npj Climate and Atmospheric Science*, 2(1):29, 2019.

[8] J. M. Gregory, T. Andrews, and P. Good. The inconstancy of the transient climate response parameter under increasing co2. *Philosophical Transactions of the Royal Society A: Mathematical, Physical and Engineering Sciences*, 373(2054):20140417, 2015.

[9] Kaoru Tachiiri, Tomohiro Hajima, and Michio Kawamiya. Increase of uncertainty in transient climate response to cumulative carbon emissions after stabilization of atmospheric co2concentration. *Environmental Research Letters*, 10(12):125018, 2015.

[10] C. Le Quéré, R. M. Andrew, J. G. Canadell, S. Sitch, J. I. Korsbakken, G. P. Peters, A. C. Manning, T. A. Boden, P. P. Tans, R. A. Houghton, R. F. Keeling, S. Alin, O. D. Andrews, P. Anthoni, L. Barbero, L. Bopp, F. Chevallier, L. P. Chini, P. Ciais, K. Currie, C. Delire, S. C. Doney, P. Friedlingstein, T. Gkritzalis, I. Harris, J. Hauck, V. Haverd, M. Hoppema, K. Klein Goldewijk, A. K. Jain, E. Kato, A. Körtzinger, P. Landschützer, N. Lefèvre, A. Lenton, S. Lienert, D. Lombardozzi, J. R. Melton, N. Metzl, F. Millero, P. M. S. Monteiro, D. R. Munro, J. E. M. S. Nabel, S. I. Nakaoka, K. O'Brien, A. Olsen, A. M. Omar, T. Ono, D. Pierrot, B. Poulter, C. Rödenbeck, J. Salisbury, U. Schuster, J. Schwinger, R. Séférian, I. Skjelvan, B. D. Stocker, A. J. Sutton, T. Takahashi, H. Tian, B. Tilbrook, I. T. van der Laan-Luijkx, G. R. van der Werf, N. Viovy, A. P. Walker, A. J. Wiltshire, and S. Zaehle. Global carbon budget 2016. *Earth Syst. Sci. Data*, 8(2):605–649, 2016.

[11] C. J. Smith, P. M. Forster, M. Allen, N. Leach, R. J. Millar, G. A. Passerello, and L. A. Regayre. Fair v1.3: a simple emissions-based impulse response and carbon cycle model. *Geosci. Model Dev.*, 11(6):2273–2297, 2018.

[12] Nathan P. Gillett, Vivek K. Arora, Damon Matthews, and M. Allen. Constraining the ratio of global warming to cumulative co2 emissions using cmip5 simulations. *Journal of Climate*, 26(18):6844–6858, 2013.

[13] Richard Millar, Myles Allen, Joeri Rogelj, and Pierre Friedlingstein. The cumulative carbon budget and its implications. *Oxford Review of Economic Policy*, 32(2):323–342, 2016.

[14] Andy Reisinger, Malte Meinshausen, and Martin Manning. Future changes in global warming potentials under representative concentration pathways. *Environmental Research Letters*, 6(2):024020, 2011.

[15] Olivier Jolliet, Assumpció Antón, Anne-Marie Boulay, Francesco Cherubini, Peter Fantke, Annie Levasseur, Thomas E. McKone, Ottar Michelsen, Llorenç Milà i Canals, Masaharu Motoshita, Stephan Pfister, Francesca Verones, Bruce Vigon, and Rolf Frischknecht. Global guidance on environmental life cycle impact assessment indicators: impacts of climate change, fine particulate matter formation, water consumption and land use. *The International Journal of Life Cycle Assessment*, 23(11):2189–2207, Nov 2018.

[16] M. Etminan, G. Myhre, E. J. Highwood, and K. P. Shine. Radiative forcing of carbon dioxide, methane, and nitrous oxide: A significant revision of the methane radiative forcing. *Geophysical Research Letters*, 43(24):12,614–12,623, 2016.

[17] G. Myhre, D. Shindell, F.-M. Bréon, W. Collins, J. Fuglestvedt, D. Huang, J.-F. Koch, D. Larmarque, B. Lee, T. Mendoza, T. Nakajima, A. Robock, G. Stephens, T. Takemura, and H. Zhang. *Anthropogenic and Natural Radiative Forcing.* Cambridge University Press, Cambridge, United Kingdom and New York, NY, USA, 2013.
